# Effectiveness of WhatsApp based debunking reminders on follow-up visit attendance for individuals with hypertension: a randomized controlled trial in India

**DOI:** 10.1186/s12889-024-19894-9

**Published:** 2024-09-09

**Authors:** Caterina Favaretti, Vasanthi Subramonia Pillai, Seema Murthy, Adithi Chandrasekar, Shirley D. Yan, Huma Sulaiman, Atul Gautam, Baljit Kaur, Mohammed K. Ali, Margaret McConnell, Nikkil Sudharsanan

**Affiliations:** 1 Professorship of Behavioral Science for Disease Prevention and Health Care, TUM School of Medicine and Health, Technical University of Munich, Munich, Germany; 2 Noora Health PLC IN, Bangalore, Karnataka India; 3 YosAID Innovation Foundation IN, Bagalore, Karnataka India; 4 Noora Health US, San Francisco, CA USA; 5 ASSRA, New Delhi, India; 6 Department of Health and Family Welfare, Government of Punjab, Chandigarh, India; 7 Hubert Department of Global Health, Rollins School of Public Health, Emory University, Atlanta, USA; 8 Department of Family and Preventive Medicine, School of Medicine, Emory University, Atlanta, USA; 9 Emory Global Diabetes Research Center, Woodruff Health Sciences Center, Emory University, Atlanta, USA; 10 Department of Global Health and Population, Harvard T.H. Chan School of Public Health, Harvard University, Boston, USA; 11 Heidelberg Institute of Global Health, Heidelberg University, Heidelberg, Germany

**Keywords:** Debunking, Health-related misconceptions, WhatsApp, Randomized Controlled Trial, India, Hypertension, Preventive care

## Abstract

**Background:**

Individuals with high blood pressure in India often miss essential follow-up visits. Missed visits contribute to gaps across the hypertension care continuum and preventable cardiovascular disease. Widespread misconceptions around hypertension care and treatment may contribute to low follow-up attendance rates, but to date, there is limited evidence of the effect of interventions to debunk such misconceptions on health-seeking behavior. We conducted a randomized controlled trial to measure whether combining information debunking commonly-held misconceptions with a standard reminder reduces missed follow-up visits among individuals with high blood pressure and investigated whether any observed effect was moderated through belief change.

**Methods:**

We recruited 388 patients with uncontrolled blood pressure from the outpatient wards of two public sub-district hospitals in Punjab, India. Participants randomly assigned to the intervention arm received two WhatsApp messages, sent 3 and 1 days before their physician-requested follow-up visit. The WhatsApp message began with a standard reminder, reminding participants of their upcoming follow-up visit and its purpose. Following the standard reminder, we included brief debunking statements aimed at acknowledging and correcting common misconceptions and misbeliefs about hypertension care seeking and treatment. Participants in the control group received usual care and did not receive any messages.

**Results:**

We did not find evidence that the enhanced WhatsApp reminders improved follow-up visit attendance (Main effect: 2.2 percentage points, p-value = 0.603), which remained low across both treatment (21.8%, 95% CI: 15.7%, 27.9%) and control groups (19.6%, 95% CI: 14.2%, 25.0%). Participants had widespread misconceptions about hypertension care but our debunking messages did not successfully correct these beliefs (p-value = 0.187).

**Conclusions:**

This study re-affirms the challenge of continuity of care for chronic diseases in India and suggests that simple phone-based health communication methods may not suffice for changing prevalent misconceptions and improving health-seeking behavior.

**Trial registration:**

The trial began on July 18th. We registered the trial on July 18th (before recruitment began), including the main outcomes, on the German Clinical Trial Register [Identifier: DRKS00029712] and published a pre-analysis plan in the Open Science Framework [osf.io/67g35].

**Supplementary Information:**

The online version contains supplementary material available at 10.1186/s12889-024-19894-9.

## Introduction

Regular follow-up visits are essential for diagnosing, treating, and controlling hypertension and other key cardiovascular disease risk factors [1]. However, in India - where 28% of adults have hypertension yet just 53% of these individuals have treated and controlled blood pressure (BP) [2] - recent studies reveal extremely low levels of follow-up visit attendance among individuals at risk of or with hypertension [3, 4]. In the Indian public healthcare context, these follow-up visits are particularly crucial as they provide an opportunity for patients to collect and refill their medication free of charge. Missed visits thus contribute substantially to gaps across the hypertension continuum and are an important target for health policy aimed at improving cardiovascular disease prevention.

Missed visits, and poor adherence in general, have significant consequences for both health and financial outcomes. For instance, a recent U.S. study found that for every 1% increase in non-adherence, the total number of cardiovascular deaths increased by 7.13 deaths per 100,000 adults, even after accounting for factors such as insurance access, education levels, income inequality, and poverty rate [5]. Financially, non-adherence results in higher healthcare costs due to more frequent hospitalizations, emergency department visits, the need for more intensive treatments, and indirect costs associated with lost productivity from premature death or disability [6]. Numerous studies have documented these impacts, emphasizing the importance of prioritizing the prevention, early detection, and effective management of hypertension [7, 8].

Many policies aimed at improving visit attendance have been designed around a hypothesis that non-attendance is accidental (i.e. due to forgetfulness or insufficient planning and organization) and can be addressed through reminder messages [9–11]. Yet, there is growing evidence that forgetfulness is not the sole driver of missed visits for asymptomatic chronic conditions in low- and middle-income countries (LMICs) [12, 13]. Individuals in India and similar contexts often hold misconceptions and misbeliefs about preventive care and treatment for conditions like hypertension. For example, our prior research in India revealed that many people mistakenly believe that once their BP returns to normal levels, they no longer need to make follow-up visits or adhere to treatment [14]. Similarly, in rural Andhra Pradesh, India, a recent evaluation of the UDAY program found that individuals at risk of hypertension or diabetes cited lack of symptoms leading to a belief that they did not require further care as a reason for not attending recommended follow-up care [15].

Recent advances in the communication sciences suggest that when individuals hold misconceptions, debunking-based communication strategies may be most effective at correcting and updating beliefs and facilitating behavior change [16]. A debunking intervention aims to acknowledge the existence of a misconception, declare it as false, and subsequently provide the correct information. Studies show that corrections are likely to be more successful when they address the misbelief with specific counter-evidence [17] and use a source of correction that is socially connected to or trusted by the individual [18, 19]. However, there is currently limited evidence of the effect of debunking strategies on chronic care-related misconceptions and behavior in a LMIC context like India.

In this study, we conducted a randomized controlled trial (RCT) of an enhanced reminder intervention among individuals with elevated BP in two primary care clinics in Punjab, India. Prior to randomization, we measured participants’ beliefs about hypertension care to assess how common misconceptions were. Our enhanced reminders combined a standard reminder about participants’ follow-up visits with debunking messages aimed at correcting common hypertension-related misconceptions. We assessed whether our enhanced reminders improved follow-up visit attendance and explored the mechanism of our intervention by also investigating the effect of debunking messages on hypertension-related knowledge and beliefs. Our main hypothesis was that bundling a traditional reminder - which addresses forgetfulness and salience bias - with debunking statements that correct misbeliefs would improve the proportion of adults who attend their physician-requested hypertension-related follow-up visits.

## Methods

### Ethics approvals and trial registration

Before recruiting participants, we received ethics approval from the ACE Independent Ethics Committee in India and the Technical University of Munich Ethics Committee in Germany. The trial began on July 18th. We registered the trial on July 18th (before recruitment began), including the main outcomes, on the German Clinical Trial Register [Identifier: DRKS00029712] and published a pre-analysis plan in the Open Science Framework [20]. Appendix Fig. 1 shows the timeline of RCT activities. We reported our study according to the CONSORT guideline (Supplemental Material). Patients were not involved in the design, or implementation, or reporting, or dissemination plans of our study. Patients received no financial or non-financial incentives to participate in the study.

### Study design, study setting, participants, and sample size

Our study was a two-arm parallel individual-level RCT. Our study took place in the outpatient primary care clinics of two sub-district hospitals in Punjab, India between July 18th, 2022, and February 20th, 2023. As public hospitals, both study sites provide free medical care to patients, including free distribution of prescribed anti-hypertensive medications onsite.

In the specific districts where our study was conducted, Sahibzada Ajit Singh Nagar and Amritsar, the hypertension prevalence in 2019 was 37.5% and 47.1%, respectively [21]. Of those with hypertension, 52.6% in Sahibzada Ajit Singh Nagar and 35.6% in Amritsar were diagnosed [21]. Among those diagnosed with hypertension, 47.7% in Sahibzada Ajit Singh Nagar and 58.2% in Amritsar were untreated. Across the care continuum, just 10.1% of individuals with hypertension in Sahibzada Ajit Singh Nagar and 7% in Amritsar had treated and controlled BP [21]. These statistics are consistent with the broader trends observed in Punjab, where the prevalence of hypertension is slightly higher than the national average in India [21]. In rural Punjab, approximately 80% of healthcare seeking occurs in private healthcare facilities [22]. Individuals often cite perceived higher quality of service and shorter waiting times for choosing private over public care [23]. In contrast, about 16% of the population opts for public healthcare providers, with 4% relying on services from the informal sector.

We recruited individuals who sought care at our study clinics and met the following inclusion criteria: older than 18 years old, able to speak and answer questions on their own in Punjabi or Hindi, have a clinician-requested follow-up visit for hypertension, have a personal phone number, and have WhatsApp installed on their smartphone. We relied on study physicians’ evaluations of whether a patient required a hypertension-related follow-up visit. Study physicians based their decisions on patients’ measured BP during the consultation and their self-reports of prior hypertension diagnosis. Nearly all the participants that clinicians requested a follow-up visit for were previously diagnosed but with uncontrolled BP (94.4% of study participants had uncontrolled BP and 97.7% reported a prior physician diagnosis). Individuals who did not meet these eligibility criteria were not invited to participate in the study. All invited individuals were asked to provide written informed consent before participation. The sample size was limited by available resources, resulting in a total of 388 participants.

### Study procedures

For each patient that attended the study clinics, physicians filled out a patient slip with information on their measured BP, whether they asked the patient to return to the clinic for a hypertension-related follow-up visit, and if so, when. In our setting, physicians most commonly request that patients with elevated BP come back after 7 days with only a minority of patients asked to come back in 14 or 30 days. Out of the 388 participants, 88.7% were instructed to come back in 7 days, 6.4% were asked to return in 14 days, and only 4.9% in 30 days. Physicians then told patients to meet with and provide the patient slip to study staff after their consultation. A study staff member then assessed patients’ eligibility for the study.

For eligible patients, study staff provided details about the study, invited patients to participate, and conducted the informed consent process. The study staff then conducted a brief baseline questionnaire, aimed at gathering participants’ demographic information (age, sex, educational level, marital status) and answers to 7 hypertension-related belief questions. These questions assessed participants’ beliefs about when to seek care, have their BP checked, and take treatment.

Approximately 40 days after each participant’s scheduled follow-up appointment, study staff conducted a phone-based endline survey with participants. We selected this timeframe to align with the clinicians’ recommendation for follow-ups within 30 days, allowing an additional 10 days for participants to attend their follow-ups. The endline survey covered two key areas: (1) the salience of the reminders and (2) responses to the same 7 hypertension-related questions asked at baseline.

### Randomization and blinding

We randomized participants to the intervention and control arms (1:1 allocation) daily (e.g. participants recruited on a given day were randomized at the end of that day) using randomly generated numbers in Stata 18. Physicians, other hospital staff, and survey enumerators were blinded to the randomized assignment. Since the study intervention was messages sent to patients, it was not possible to blind participants to the intervention assignment.

### Intervention

Our intervention was two WhatsApp messages sent to intervention-group participants 3 and 1 days before their physician-requested follow-up visit. The WhatsApp message began with a standard reminder that reminded participants about their upcoming follow-up visit and the purpose of the visit. Below the standard reminder component, we included brief debunking statements that sought to acknowledge and correct common misconceptions and misbeliefs about hypertension. Our messages targeted two key misconceptions: (1) that hypertension care is only needed when one feels symptoms and (2) that hypertension care is not required if individuals’ BP reaches a controlled range (< 140/90mmHg). We selected these misconceptions and misbeliefs to target based on our prior studies of hypertension and prior research on diabetes care-seeking in India [14, 15]. For content, the messages first acknowledged the incorrect beliefs, stated that they were false, and lastly provided the correct factual information. In a close partnership with the Government of Punjab, Noora Health’s implementing partners in India sent the WhatsApp messages to participants. This decision was made to enhance trust and credibility in the reminders, aligning with recommendations for improving the effectiveness of communication and debunking efforts [18, 19]. The exact content of the WhatsApp reminders is shown in Appendix Table 1.

We sent the WhatsApp reminders through the *Text.it* platform [24]. The platform allows for semi-automated message sending and provides backend information on whether the messages were successfully delivered to individuals’ phones.

We chose WhatsApp for delivering our enhanced reminders because it is the main platform for phone-based personal communication in India, making it a more salient and engaging platform than standard SMS messages. Recent data shows that WhatsApp has become India’s most-used social media app, with users spending an average of 20 h per month on the platform in 2021 [25]. This widespread usage makes WhatsApp more effective for our reminders compared to standard SMS, which is mostly used by companies, government entities, and for spam in India, and thus often ignored by recipients.

### Outcomes

Our main outcome was attendance of the physician-requested follow-up visit. We measured attendance through electronic attendance records maintained by the study staff. In line with our pre-analysis plan, we created a binary indicator for having attended the follow-up visit if a participant revisited the clinic after receiving the first reminder and within 7 days of their requested follow-up visit date. In supplementary analyses, we also investigated follow-up visit attendance for up to 30 days after their requested date.

Our secondary outcome was an index of hypertension misconceptions. We constructed this outcome as a count of the number of the 7 hypertension belief questions that individuals provided incorrect answers for (range 0–7, with 7 indicating the greatest degree of misconceptions). We generated this outcome using participants’ responses to the endline questions on hypertension beliefs; however, in sensitivity analyses, we also included the index based on participant responses collected in person on the recruitment day, before randomization, to increase the accuracy of the estimates. These questions had open-ended responses and we did not provide participants with answers to choose from. After the study, we classified the open-ended responses into discrete response groups to determine whether individuals’ beliefs were correct or inaccurate.

### Other variables

Analyses also incorporated individuals’ self-reported age (categorized as either over or younger than the mean of 48 years old), sex (male or female), level of completed education (higher than secondary education or lower), and their measured baseline BP. Baseline BP was classified as either controlled or stage 1 hypertension (systolic less or equal to 159 mmHg, or, diastolic less or equal than 99 mmHg) versus stage 2 hypertension (systolic more or equal to 160 mmHg, or, diastolic more or equal than 100 mmHg). All demographic variables were based on self-reported information collected during the baseline survey. Baseline BP values were drawn from the patient slip given to each participant during the consultation; BP was measured by the facility nurse before individuals consulted with the physician.

### Statistical methods

We first present descriptive characteristics of the sample stratified by the intervention assignment to assess covariate balance. We used chi-squared and two-sample t-tests to test for differences in the baseline characteristics across intervention and control groups.

Next, we described the prevalence of hypertension-related misconceptions among participants by presenting the proportion of participants who gave an incorrect answer to each of the 7 hypertension-related belief questions at baseline.

We estimated the intention-to-treat (ITT) effect of being assigned to receive the reminder message on follow-up visit attendance through a linear probability model with attendance of the visit as the dependent variable, treatment assignment as the main independent variable, and a facility fixed effect. We expressed the treatment effect on the probability difference scale. We similarly estimated the ITT effect of the intervention on hypertension-related misconceptions using linear regression models with the hypertension misconception index as the main dependent variable.

Next, we estimated whether the treatment effects on the main and secondary outcome differed by age, sex, education, baseline BP, and baseline misconception score through regression models that additionally included an interaction between the treatment assignment variable and the heterogeneity variable (separate regressions for each outcome and heterogeneity variable).

Finally, we estimated treatment on treated (ToT) effects using an instrumental variables approach [26]. Specifically, we used the random assignment to intervention or control as an instrument for whether an individual received the enhanced WhatsApp reminder - measured through backend data from the *Text.it* platform - using two-stage least-squares regression models. In contrast to the ITT effects, these effects represent the effect of receiving the enhanced WhatsApp message and thus account for the fact that not all individuals assigned to receive reminders had successfully delivered messages.

We conducted all analyses in Stata 18 and R version 4.2.3.

## Results

### Descriptive characteristics and balance

**Fig. 1:**
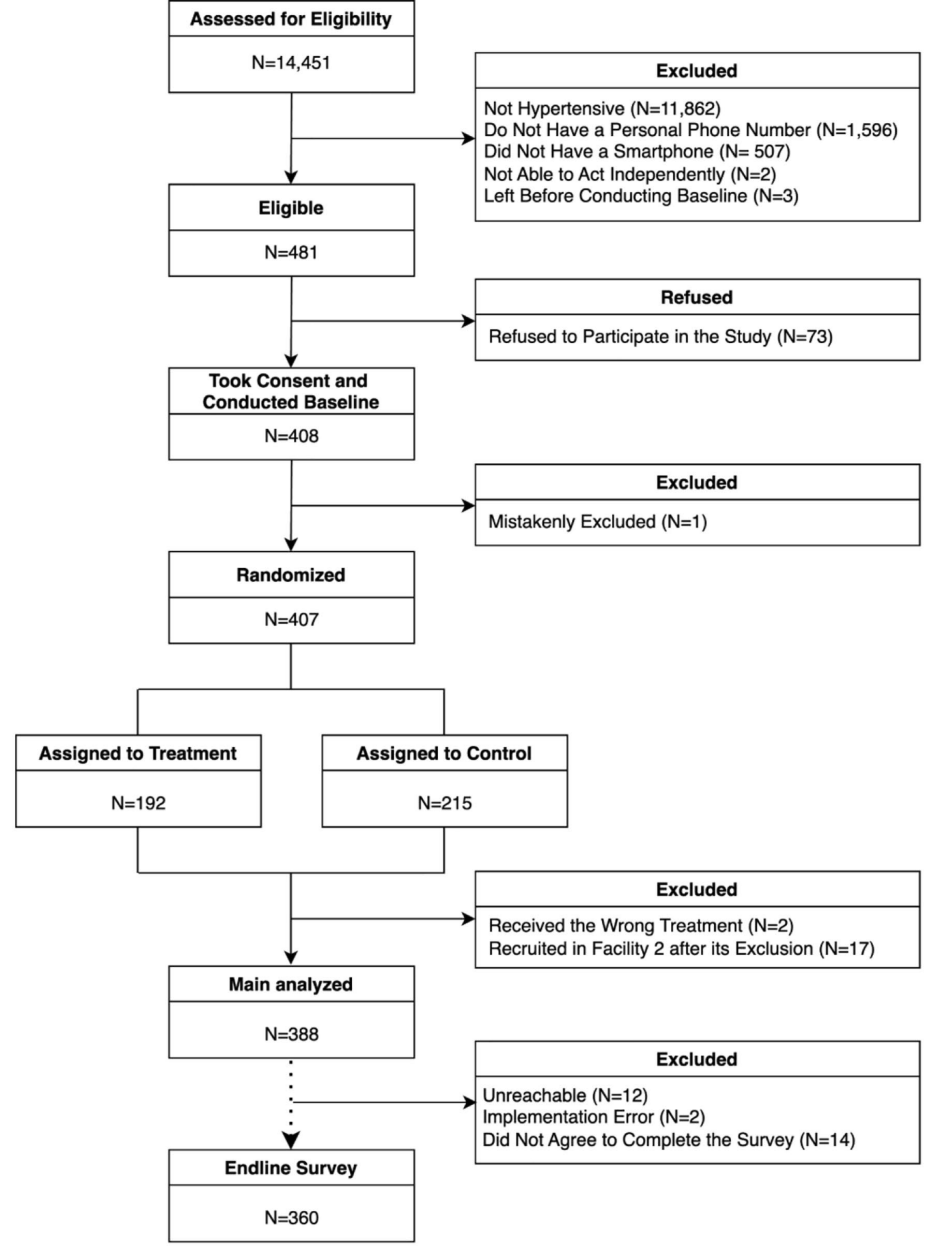
CONSORT Diagram

Study staff met with 14,451 patients over the study period, of which 2,589 had uncontrolled BP. 481 met the eligibility criteria, and among those that did, 408 agreed to participate (Fig. 1). We erroneously excluded 1 (0.2%) participant before randomization, 2 (0.5%) because they received the wrong treatment due to an operational error, and 17 (4.2%) due to a data collection stop in the second facility after a change in staffing. The final sample consisted of 388 participants.

Participants’ average age was 48.7 years (SD = 11.4) and 66.5% (*N* = 258) were female (Table 1). Most participants had completed secondary school (48.4%, *N* = 188) and nearly all were married (98.2%, *N* = 381). The mean baseline systolic BP was 158.9 mmHg (SD = 18.5) and the mean diastolic BP was 96.2 mmHg (SD = 12.2). We found no evidence of imbalance between the intervention and control groups.

**Table 1:** Baseline characteristics and randomization balance, *N* = 388, Punjab, India

	Study Sample			
	Overall	Treatment Arm	Control Arm	
	*N* = 388	*N* = 179	*N* = 209	*p*-value
**Age (mean**,** SD)**	48.7 (11.4)	48.7 (11.2)	48.6 (11.6)	0.941
**Sex (n**,**%)**				
Male	130 (33.5)	56 (31.3)	74 (35.4)	0.391
Female	258 (66.5)	123 (68.7)	135 (64.6)	
**Education (n**,**%)**				
Have not attended school	6 (1.5)	3 (1.7)	3 (1.4)	0.526
Less than secondary school	94 (24.2)	38 (21.2)	56 (26.8)	
Secondary school completed	188 (48.4)	89 (49.7)	99 (47.4)	
Higher secondary school completed	76 (19.6)	38 (21.2)	38 (18.2)	
Diploma or higher	24 (6.2)	11 (6.1)	13 (6.2)	
**Marital Status (n**,**%)**				
Unmarried	6 (1.5)	4 (2.2)	2 (0.9)	0.391
Married	381 (98.2)	175 (97.8)	206 (98.6)	
Widowed	1 (0.3)	0 (0.0)	1 (0.5)	
**Baseline Blood Pressure (mean**,** SD)**				
Systolic	158.9 (18.5)	158.1 (17.9)	159.7 (19.1)	0.416
Diastolic	96.3 (12.2)	95.9 (11.5)	96.5 (12.7)	0.632
**Baseline Misconception Score (mean**,** SD)**	2.13 (1.65)	2.14 (1.67)	2.13 (1.64)	0.901

*Notes*: p-Values for sex, education, marital status correspond to a chi-square test and t-test for age, baseline blood pressure values and misconception score. Blood pressure readings were available for 373 participants (96.1%)

### Baseline hypertension-related misbeliefs

**Fig. 2:**
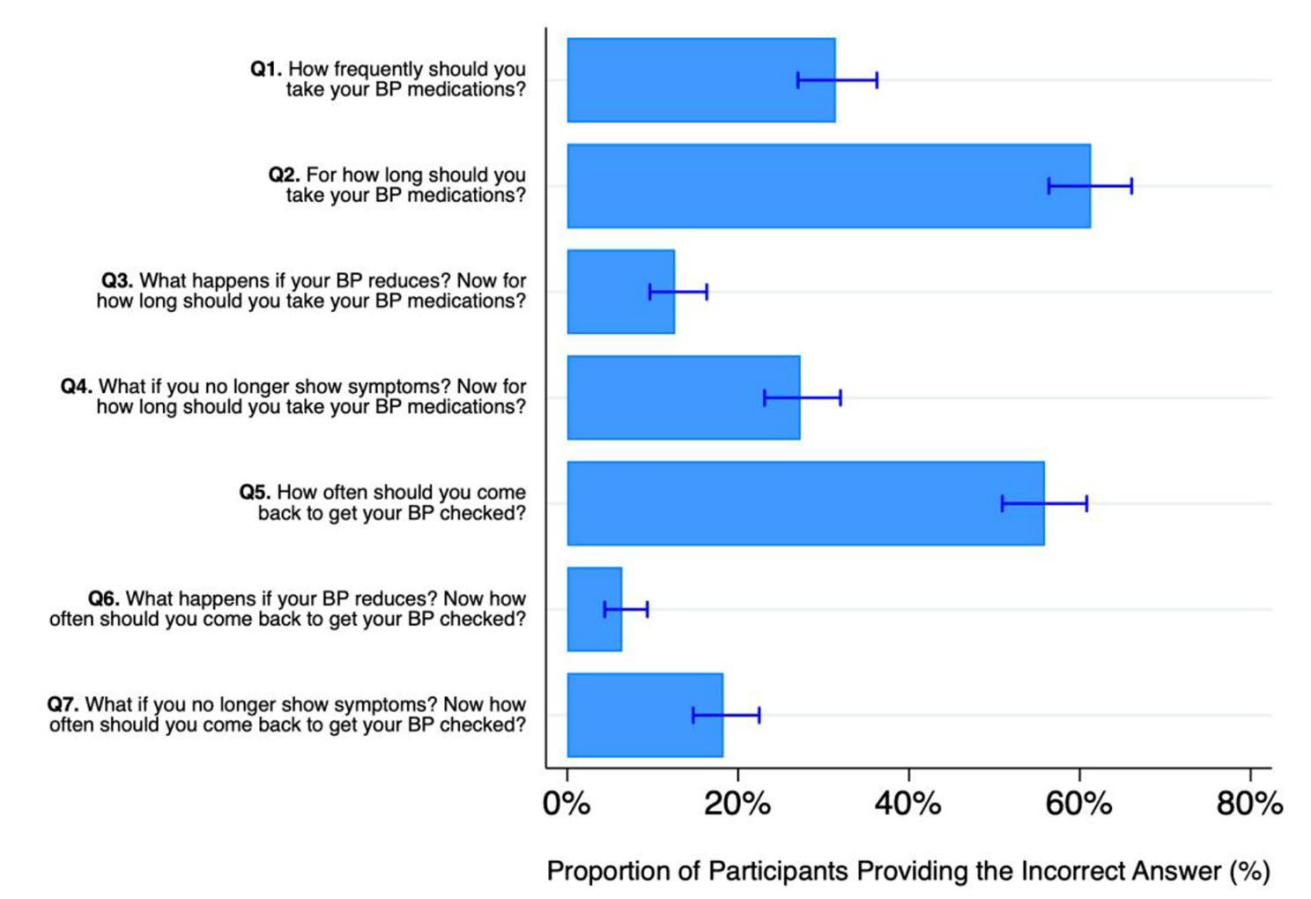
Percentage of participants who stated inaccurate beliefs about hypertension across seven belief questions, *N* = 388, Punjab, India

We found evidence of highly prevalent misconceptions about hypertension care and treatment (Fig. 2). 55.9% (*N* = 217, 95% CI: 50.9%, 60.8%) of participants had misconceptions about how often they should seek care to have their BP checked. Similarly, 61.3% (*N* = 238, 95% CI: 56.4%, 66.1%) of participants had misconceptions about how long they should take their hypertension treatment, and 31.4% (*N* = 122, 95%: 27.0%, 36.2%) about how frequently they should take medicines. Across questions, participants had misconceptions for 2.13 (SD = 1.65) of the 7 hypertension-related belief questions on average. The exact responses to each question are presented in Appendix Table 2.

### Main Treatment effects

**Fig. 3:**
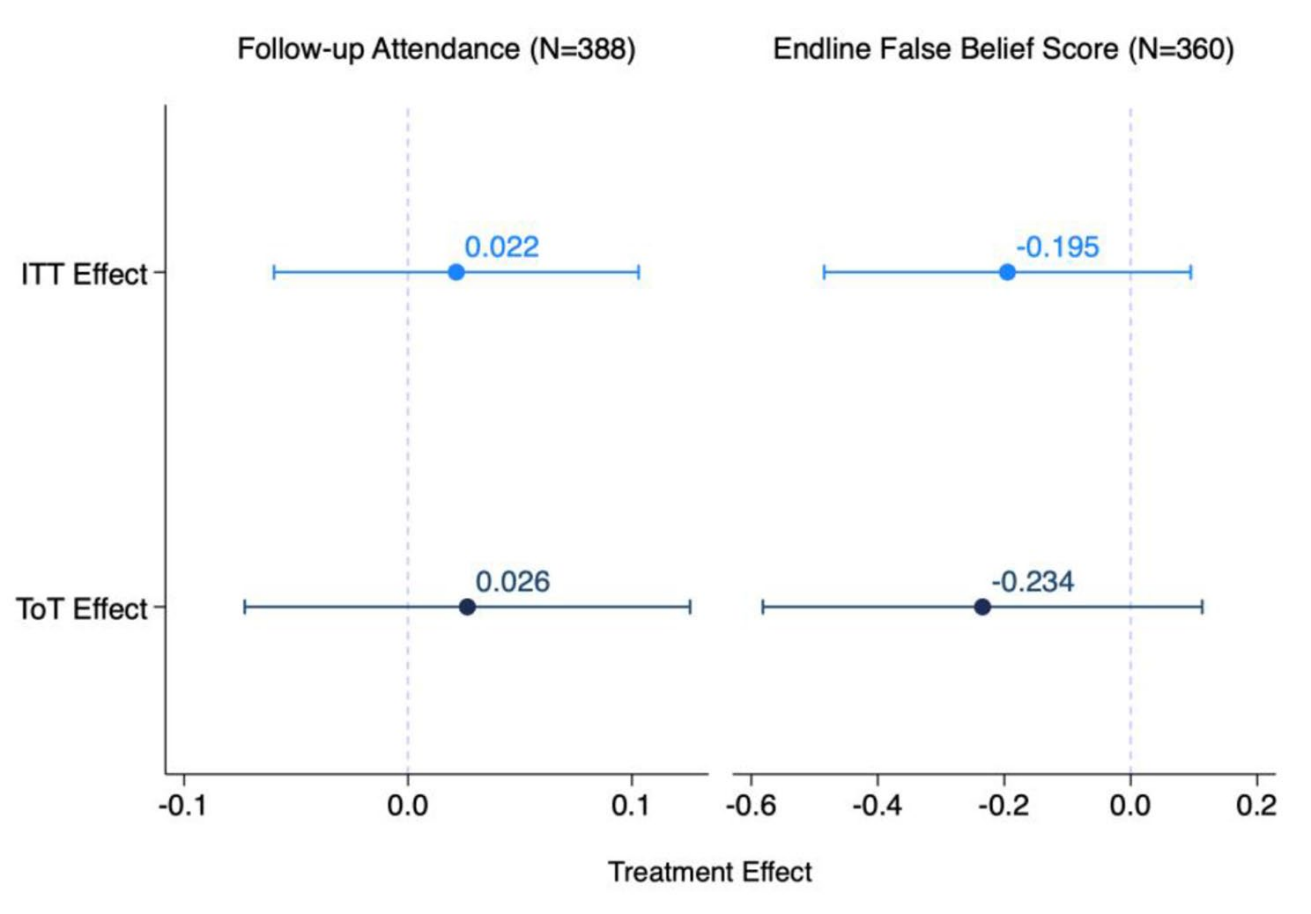
Intention to treat and treatment on treated enhanced reminder effects on follow-up attendance and hypertension-related misconceptions, Punjab, India. *Notes*: Follow-up attendance takes on the values 1 (attended) or 0 (not attended), with related coefficients expressed as percentage points. Misconception score ranges from 0 to 7 and the corresponding coefficients are in units. Of the 388 participants composing the final sample, 360 (92.8%) provided answers to the 7 hypertension-related questions. Of the remaining 28, 12 did not pick up the phone, 2 participants were not contacted due to an implementation error, and 14 did not complete the survey. Both models include facility fixed effects. Error bars represent 95% confidence intervals

We did not find evidence that the enhanced WhatsApp reminders improved follow-up visit attendance among the overall sample (Fig. 3). Our message had a non-significant 2.2 percentage point (95% CI: -6.0%, 10.3%; *p* = 0.603) effect on follow-up compared to the control group follow-up percentage of 19.6% (95% CI: 14.2%, 25.0%).

Similarly, we did not find evidence that the debunking messages in the WhatsApp reminders improved hypertension-related beliefs and knowledge. Individuals in the treatment group had a mean misconception index that was 0.195 units (95% CI, -0.48, 0.10; *p* = 0.187) lower than the control group’s mean of 2.101 (95% CI: 1.88, 2.33). This null result remained in regression specifications that examined the change in misconceptions between endline and baseline as the dependent variable and that adjusted for the baseline misconception score as a covariate (Appendix Table 3).

### Differences in the treatment effects by age, sex, education, baseline blood pressure, and baseline misconception score

**Fig. 4:**
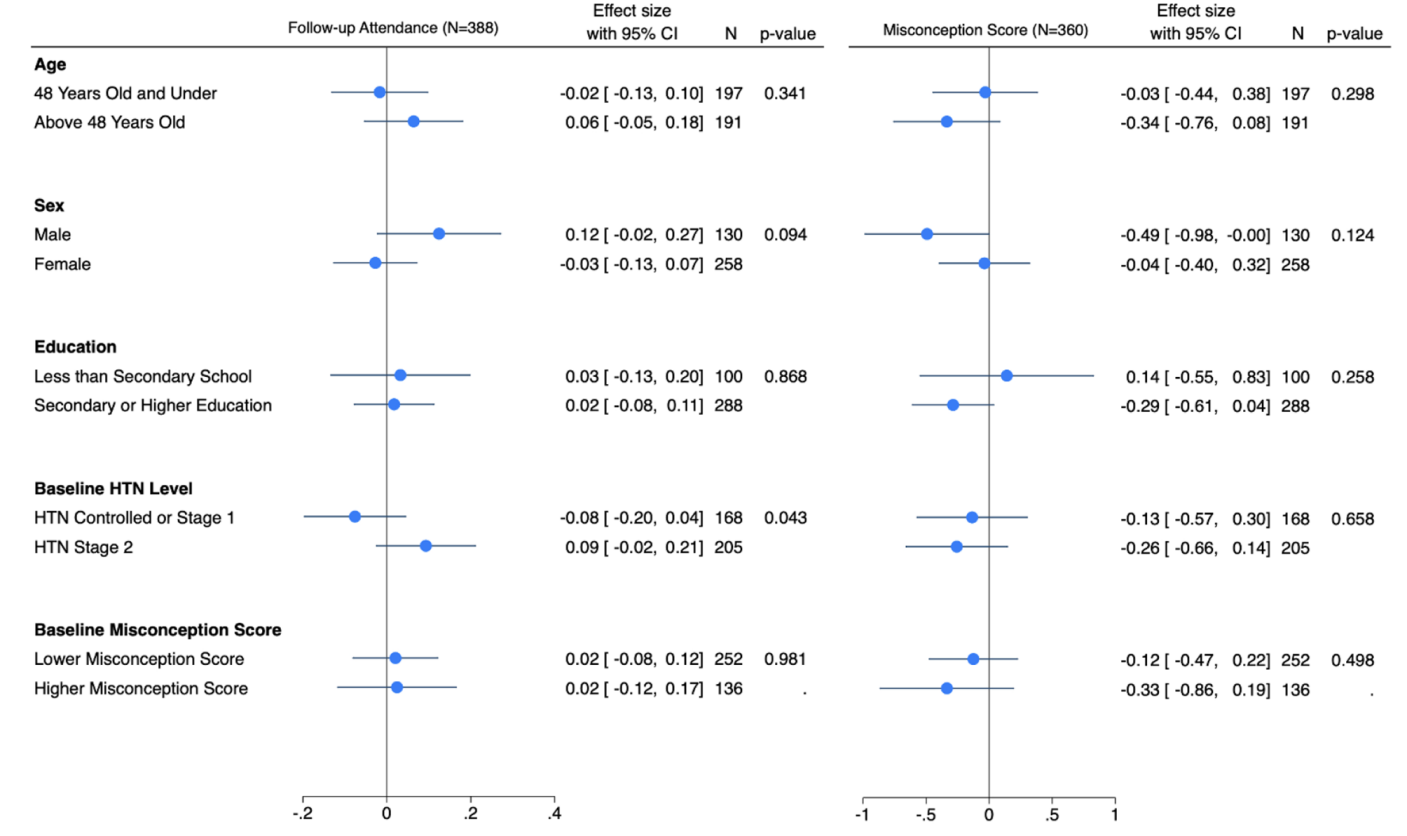
Differences in the reminder effect on primary and secondary outcomes by age, sex, education, baseline hypertension level, and baseline misconception score, Punjab, India. *Notes*: Follow-up attendance takes on the values 1 (attended) or 0 (not attended), with related coefficients expressed as percentage points. Controlled or stage 1 hypertension indicates systolic BP < = 159 mmHg or diastolic BP < = 99 mmHg and hypertension stage 2 indicates systolic BP > = 160 mmHg or diastolic BP > = 100 mmHg. Misconception score ranges from 0 to 7 and the corresponding coefficients are in units. Of the 388 participants composing the final sample, 360 (92.8%) provided answers to the 7 hypertension-related questions. Of the remaining 28, 12 did not pick up the phone, 2 participants were not contacted due to an implementation error, and 14 did not complete the survey. All models include facility fixed effects. The x-axis displays reminder effects within each subgroup, with error bars representing 95% confidence intervals

We observed that the effect of the enhanced reminder on follow-up visit attendance may vary by sex and baseline hypertension level, although these results were not statistically significant (Fig. 4). For men, the enhanced reminder improved follow-up visit attendance by 12.0% points (95% CI: -2.0%, 27.0%), while for women, there was a decrease of 3.0% points (95% CI: -13.0%, 7.0%) (difference 95% CI: -32.6%; 2.6%; difference p-value: 0.094). For participants with hypertension stage 2, the enhanced reminder increased follow-up attendance by 9.0% points (95% CI: -2.4%, 21.1%), while for those with controlled or hypertension stage 1, there was a decrease of 8.0% points (95% CI: -19.6%, 4.4%) (difference 95% CI: 0.5%, 34.1%; difference p-value: 0.043). These directional findings suggest scope for further studies to explore these patterns in more detail. We do not find evidence of any treatment effect differences by age and education for the main outcome and no evidence of treatment heterogeneities across any of the variables on hypertension-related misbeliefs.

### Treatment on treated effects

Although 18.4% (*N* = 33) of the enhanced reminders were not successfully delivered to participants’ phones due to spam filters or invalid phone numbers, we do not find evidence that the previous null effects were driven by non-delivery (Fig. 3). We found null treatment on the treated intervention effects on follow-up visit attendance (ToT effect: 2.6 percentage points, *p* = 0.602) and hypertension-related misbeliefs (ToT effect: -0.234, p-value = 0.186).

### Robustness and sensitivity

Our conclusions remained unchanged when we extended the time horizon for the main outcome to having attended the clinic within 14 and 30 days - rather than 7 days (Appendix Tables 4 and 5). Our conclusions also remained the same when we estimated models that additionally included baseline covariates and when using logistic rather than linear probability models (Appendix Tables 6, 7 and 8). To account for variation attributable to different physicians, we repeated the analysis including fixed effects for the surveyor who recorded patient interactions with the physician each day. As each physician’s consultations were observed by a single surveyor, this effectively acted as physician-fixed effects, and the results remained consistent (Appendix Table 9, and 10). We also examined the effect of the enhanced reminder on each of the seven misconception questions separately (Appendix Table 11). Notably, we found a significant improvement in the accuracy of responses to only one question, which asked how frequently you should take your anti-hypertensive medications. Participants assigned to receive the enhanced reminder were 11.4% points more likely to provide the correct answer compared to those in the control group (95% CI: 2.73–20.2%; p-value = 0.010). In contrast, the remaining six questions did not show statistically significant effects from the enhanced reminder.

## Discussion

In this randomized trial, we found that WhatsApp messages that combined a traditional reminder with messages debunking hypertension-related misbeliefs and misconceptions did not improve follow-up attendance among individuals with elevated BP in Punjab, India. Among individuals in both the intervention and control groups, only about 21% attended their hypertension-related follow-up visit. One important potential contributor to these low follow-up attendance rates was widespread misconceptions about when hypertension care and treatment are required. Over 78% of individuals in our study believed that hypertension care was only required when they felt symptoms, or, that they could stop care-seeking and treatment when their symptoms resolved. Our enhanced reminder was successful in correcting one specific misconception regarding how often they should take their medication. However, it did not manage to address other critical areas of misbelief. Specifically, participants still had misconceptions about how long they should continue using their medication, the recommended frequency of follow-up visits, and the appropriate actions to take when their symptoms disappear or their readings return to normal. Due to these persistent misconceptions, we cannot conclusively claim that our intervention effectively corrected participants’ overall misconceptions about hypertension care. Our study re-affirms the important challenge of improving continuity of care for non-communicable diseases in India. More broadly, our findings reveal that simple text-based health communication methods may not be sufficient for changing prevalent misconceptions and improving health-seeking behavior.

Our study design was motivated by evidence that when individuals hold misconceptions, communication strategies that use debunking may be more effective at changing beliefs than simply providing individuals with factual information [16, 27]. It remains unclear why debunking was not successful in our context. The first possible explanation is that while debunking has been proposed as a communication strategy, there is little empirical evidence of its effectiveness in changing health misconceptions in an LMIC context like India. Debunking may not be effective in our context, where other communication strategies may be more promising. Second, how effective communication interventions are is tightly tied to their intensity and salience [28]. While 62.9% of participants recalled receiving the two WhatsApp message reminders at endline and 88.9% correctly recognized their purpose, the relatively low intensity of our intervention might have diminished its impact. More intensive debunking approaches that use additional messages [29], interactive messages [30], or more personal communication mediums such as phone calls [28] and in-person discussions [31] may be more effective at changing beliefs than passive one-way phone messages.

Research from psychology highlights that changing beliefs is more challenging when those beliefs arise from inaccurate mental models [16, 32]. Our findings suggest that this may be the case in the Indian context: individuals may be incorrectly applying a mental model of healthcare seeking based on their experiences with acute care for infectious conditions to preventive care for non-communicable diseases. For example, individuals use the presence of symptoms as a cue for when they require care and the resolution of symptoms as a heuristic that care is no longer needed. In such contexts, debunking alone may not be sufficient to change beliefs. Rather, individuals may need to be given a convincing alternative mental model that helps them understand why their initial beliefs were not correct. Overall, our results should not be interpreted as evidence against debunking in general, but rather that short debunking statements delivered through mobile-phone messages may not be sufficiently salient to impact deeply rooted beliefs.

Our results also contribute to the broader literature on the effectiveness of phone-based reminders on health-seeking behavior. Overall, the existing literature on reminders is highly mixed, with several studies finding null or small effects [10, 11]. For example, recent mega studies have tested and compared the effect of over 20 SMS-delivered messages on a range of preventive health-seeking behaviors, including vaccinations against influenza [33, 34] and COVID-19 [35]. A major conclusion of these studies is that most messages are not successful at improving health-seeking behavior for preventive care, and even the messages that are successful often have modest effects. Our finding of no effect is thus consistent with prior research and raises the important question of when reminders are effective. One hypothesis supported by our study is that reminders are less effective in contexts where people hold misconceptions that lead them to believe that they do not require preventive care. Reminders may thus be effective in contexts without widespread misconceptions or when combined with more effective strategies for changing beliefs.

Our study has several important limitations. First, we were only able to recruit 388 individuals due to budget limitations and were therefore only powered to detect a minimum effect size of 13 percentage points. Second, since our study was conducted in two sites, our results may not generalize to a broader population. However, it is unclear how reminder or debunking effects may have been different in other populations.

Overall we found very low levels of follow-up attendance for essential hypertension visits among a sample of individuals with poorly controlled hypertension in Punjab, India. Among this population, misconceptions about hypertension were common. We found limited evidence that these misconceptions can be debunked by a short WhatsApp intervention. Exploring alternative ways of changing deeply rooted beliefs about preventive care in India is an important step for improving the treatment and control of hypertension and related chronic conditions among the rapidly growing population of older adults in the country.

## Electronic supplementary material

Below is the link to the electronic supplementary material.

Supplementary Material 1

Supplementary Material 2

## Data Availability

The datasets generated and analyzed during the current study are available from the corresponding author upon reasonable request.

## Abbreviations

BP: Blood Pressure
RCT: Randomized Controlled Trial
LMIC: Low- and Middle-Income Country
ITT: Intention-To-Treat
ToT: Treatment on Treated
CI: Confidence Interval
SD: Standard Deviation
