# Supplementary material for: Effectiveness of WhatsApp based debunking reminders on follow-up visit attendance for individuals with hypertension: a randomized controlled trial in India
Source: BMC Public Health. 2024 Sep 9;24:2441. doi: 10.1186/s12889-024-19894-9 (PMC11382525; doi:10.1186/s12889-024-19894-9)
Supplement: Supplementary file 2 — Supplementary Material 2 [file 12889_2024_19894_MOESM2_ESM.docx]

**Effectiveness of WhatsApp based debunking reminders on follow-up visit attendance for individuals with hypertension: A randomized controlled trial**

Caterina Favaretti^1^, M.Sc.; Vasanthi Subramonia Pillai^1^, M.Sc.; Seema Murthy^2^, MPH; Adithi Chandrasekar^3^, MPH; Shirley D. Yan^4^, MSPH; Huma Sulaiman^3^, M.Sc.; Atul Gautam^5^, M.Sc.; Baljit Kaur^6^, M.Sc.; Mohammed K. Ali^7,8,9^, PhD; Margaret McConnell^10^, PhD; Nikkil Sudharsanan^1,11^, PhD

**Appendix**

[**Figure A1 - Trial Timeline 2**](#_4r7lp1as0jpc)

[**Table A1 - WhatsApp Reminders 2**](#_ucw36qd9qvro)

[**Table A2 - Participants’ answers to each false belief question at baseline, N=388, Punjab, India 3**](#_auuw8nzfss8c)

[**Table A3 - Intention to treat and treatment on treated reminder effects on endline misconception score, Punjab, N=360, India 4**](#_y0gbtbqt45we)

[**Table A4 - Intention to treat and treatment on treated reminder effects on follow-up attendance (14-day window), N=388, Punjab, India 4**](#_v29wp9rfm7x0)

[**Table A5 - Intention to treat and treatment on treated reminder effects on follow-up attendance (30-day window), N=388, Punjab, India 5**](#_4rrts4lmd5pw)

[**Table A6 - Robustness of intention to treat and treatment on treated reminder effects on follow-up attendance to the inclusion of covariates, N=388, Punjab, India 5**](#_r68cms1p1jlt)

[**Table A7 - Robustness of intention to treat and treatment on treated reminder effects on endline misconception score to the inclusion of covariates, N=360, Punjab, India 6**](#_496c8eijq5q)

[**Table A8 - Intention to treat and treatment on treated reminder effects on follow-up attendance using logistic rather than linear probability regression models, N=388, Punjab, India 6**](#_u0onf3m8xrpc)

[**Table A9 - Robustness of intention to treat and treatment on treated reminder effects on follow-up attendance to the inclusion of surveyors fixed effect, N=373, Punjab, India 7**](#_qao6xtmm942v)

[**Table A10 - Robustness of intention to treat and treatment on treated reminder effects on endline misconception score to the inclusion of surveyors fixed effect, N=346, Punjab, India 7**](#_a958hrvdxynn)

[**Table A11 - Intention to treat and treatment on treated reminder effects on each question composing the endline misconception score, Punjab, N=360, India 8**](#_ep0ldv8mksaj)

#

#

# Figure A1 - Trial Timeline


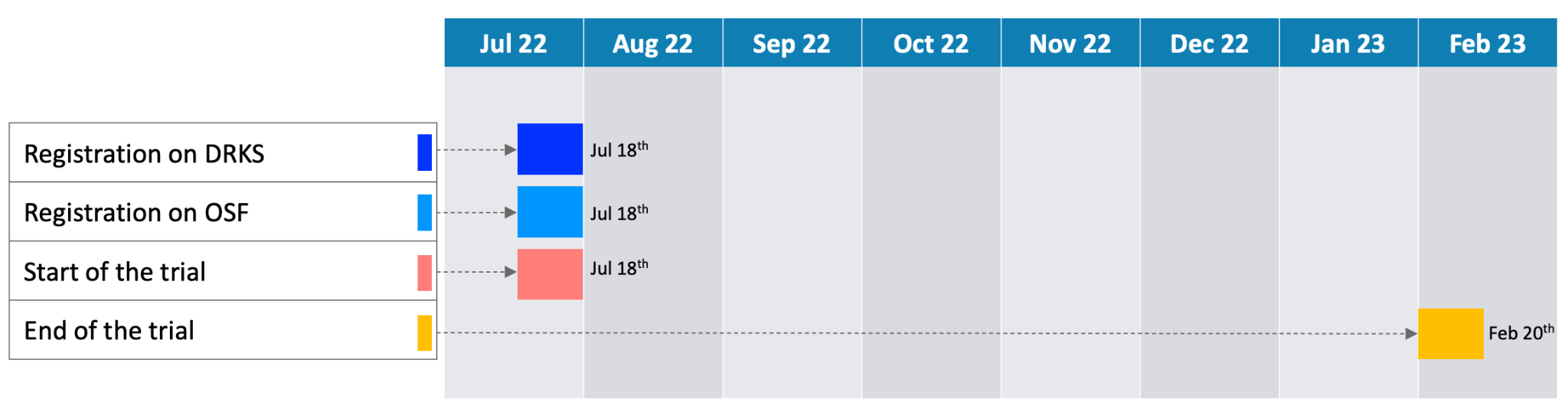


# Table A1 - WhatsApp Reminders

| **Timing** | **Content** |
| --- | --- |
| 3 days before the appointment | “Hello [Name], we are messaging regarding your visit to the [Location] SDH and the study we had discussed with you by the Gov’t of Punjab. We just wanted to remind you that you should come back to SDH on [Date] to refill your medications and get your regular blood pressure check.  Many people are surprised to know this, but you need to see the doctor for regular visits and may need medications and care even if your blood pressure has come back down to normal or you no longer feel symptoms such as headaches or gidiness. You will need to take medicines as long as the doctor has advised and pick-up your current medicines if you have finished them.” |
| 1 day before the appointment | “Hello [Name], we are sending one last reminder from [Location] SDH to visit the hospital again to refill your medications and your blood pressure check.  If you have already gone, great job! If you haven’t yet, remember that BP is different from other conditions and that you require regular check-ups and medications even if you do not feel unwell or your blood pressure has returned to normal.” |

#

# Table A2 - Participants’ answers to each false belief question at baseline, N=388, Punjab, India

| **Question** | **Answer** | **N (%)** |
| --- | --- | --- |
| 1. How frequently should you take your BP medications? | a. Daily | 205 (52.8) |
|  | **b. A couple of times a week** | 59 (15.2) |
|  | **c. Until their symptoms stop** | 63 (16.2) |
|  | d. As often as their doctor advises | 61 (15.7) |
| 2. For how long should you take your BP medications? | **a. Till I feel better** | 102 (26.3) |
|  | **b. Till my BP is normal** | 111 (28.6) |
|  | c. As per my doctor’s advice | 115 (29.6) |
|  | **d. For a few years** | 25 (6.4) |
|  | e. Forever | 35 (9.0) |
| 3. What happens if your BP reduces? Now for how long should you take your BP medications? | a. I need to keep on taking medications | 141 (36.3) |
|  | b. I should take medications for as long as my doctor advises | 198 (51.0) |
|  | **c. I can stop taking medications** | 49 (12.6) |
| 4. What if you no longer show symptoms? Now for how long should you take your BP medications? | a. I need to keep on taking medications | 120 (30.9 |
|  | b. I should take medications for as long as my doctor advises | 162 (41.7) |
|  | **c. I can stop taking medications** | 106 (27.3) |
| 5. How often should you come back to get your BP checked? | a. Weekly | 114 (29.4) |
|  | b. Monthly | 11 (2.8) |
|  | **c. Only when I feel ill** | 198 (51.0) |
|  | d. A couple times a year | 0 (0.0) |
|  | **e. Until my symptoms stop** | 6 (1.5) |
|  | **f. Until my BP reaches control** | 13 (3.3) |
|  | g. As often as my doctor advises | 46 (11.9) |
| 6. What happens if your BP reduces? Now how often should you come back to get your BP checked? | a. I need to keep on getting my BP checked | 186 (47.9) |
|  | b. I should get my BP checked for as long as my doctor advises | 177 (45.6) |
|  | **c. I can stop getting my BP checked** | 25 (6.4) |
| 7. What if you no longer show symptoms? Now how often should you come back to get your BP checked? | a. I need to keep on getting my BP checked | 176 (45.4) |
|  | b. I should get my BP checked for as long as my doctor advises | 141 (36.3) |
|  | **c. I can stop getting my BP checked** | 71 (18.3) |

*Notes*: The incorrect answers are highlighted in bold.

# Table A3 - Intention to treat and treatment on treated reminder effects on endline misconception score, Punjab, N=360, India

|  | **Misconception Score** | | | |
| --- | --- | --- | --- | --- |
|  | **ITT** | | **ToT** | |
|  | (1) | (2) | (3) | (4) |
| **Reminder Effect** | -0.195  (0.147) | -0.194  (0.148) | -0.234  (0.177) | -0.233  (0.177) |
| **Facility Fixed Effect** | YES | YES | YES | YES |
| **False Misconception Score at Baseline** | NO | YES | NO | YES |
| **Control Mean** | 2.101 | 2.101 | 2.101 | 2.101 |
| **N** | 360 | 360 | 360 | 360 |

*Notes*: Misconception score ranges from 0 to 7 and the corresponding coefficients are in units. Of the 388 participants composing the final sample, 386 were contacted to conduct the endline survey. Due to an implementation mistake, we failed to contact 2 (0.5%) participants. Of the remaining 386, 12 never picked up the phone (3.1%) and 14 (3.6%) ended the call before answering the seven false belief questions. Robust standard errors in parentheses. * p<0.1, ** p<0.05, ***p<0.01.

# Table A4 - Intention to treat and treatment on treated reminder effects on follow-up attendance (14-day window), N=388, Punjab, India

|  | **Follow-up Attendance** | |
| --- | --- | --- |
|  | **ITT** | **ToT** |
|  | (1) | (2) |
| **Reminder Effect** | -0.011  (0.043) | -0.014  (0.052) |
| **Facility Fixed Effect** | YES | YES |
| **Control Mean** | 0.234 | 0.234 |
| **N** | 388 | 388 |

*Notes*: Coefficients expressed as percentage points. Robust standard errors in parentheses. * p<0.1, ** p<0.05, ***p<0.01.

#

# Table A5 - Intention to treat and treatment on treated reminder effects on follow-up attendance (30-day window), N=388, Punjab, India

|  | **Follow-up Attendance** | |
| --- | --- | --- |
|  | **ITT** | **ToT** |
|  | (1) | (2) |
| **Reminder Effect** | 0.014  (0.046) | 0.017  (0.056) |
| **Facility Fixed Effect** | YES | YES |
| **Control Mean** | 0.277 | 0.277 |
| **N** | 388 | 388 |

*Notes*: Coefficients expressed as percentage points. Robust standard errors in parentheses. * p<0.1, ** p<0.05, ***p<0.01.

# Table A6 - Robustness of intention to treat and treatment on treated reminder effects on follow-up attendance to the inclusion of covariates, N=388, Punjab, India

|  | **Follow-up Attendance** | | | |
| --- | --- | --- | --- | --- |
|  | **ITT** | | **ToT** | |
|  | (1) | (2) | (3) | (4) |
| **Reminder Effect** | 0.022  (0.041) | 0.022  (0.043) | 0.026  (0.051) | 0.028  (0.052) |
| **Facility Fixed Effect** | YES | YES | YES | YES |
| **Covariates** | NO | YES | NO | YES |
| **Control Mean** | 0.196 | 0.196 | 0.196 | 0.196 |
| **N** | 388 | 388 | 388 | 388 |

*Notes*: Covariates include age, sex, and education, and coefficients are expressed as percentage points. Robust standard errors in parentheses. * p<0.1, ** p<0.05, ***p<0.01.

#

# Table A7 - Robustness of intention to treat and treatment on treated reminder effects on endline misconception score to the inclusion of covariates, N=360, Punjab, India

|  | **Misconception Score** | | | |
| --- | --- | --- | --- | --- |
|  | **ITT** | | **ToT** | |
|  | (1) | (2) | (4) | (5) |
| **Reminder Effect** | -0.194  (0.148) | -0.191  (0.150) | -0.233  (0.177) | -0.230  (0.180) |
| **Facility Fixed Effect** | YES | YES | YES | YES |
| **Misconception Score at Baseline** | YES | YES | YES | YES |
| **Covariates** | NO | YES | NO | YES |
| **Control Mean** | 2.101 | 2.101 | 2.101 | 2.101 |
| **N** | 360 | 360 | 360 | 360 |

*Notes*: Covariates include age, sex, and education. Robust standard errors in parentheses. * p<0.1, ** p<0.05, ***p<0.01.

# Table A8 - Intention to treat and treatment on treated reminder effects on follow-up attendance using logistic rather than linear probability regression models, N=388, Punjab, India

|  | **Follow-up Attendance** | |
| --- | --- | --- |
|  | **ITT** | **ToT** |
|  | (1) | (2) |
| **Reminder Effect** | 0.021  (0.041) | 0.026  (0.050) |
| **Facility Fixed Effect** | YES | YES |
| **Control Mean** | 0.196 | 0.196 |
| **N** | 388 | 388 |

*Notes*: Robust standard errors in parentheses. * p<0.1, ** p<0.05, ***p<0.01.

#

# Table A9 - Robustness of intention to treat and treatment on treated reminder effects on follow-up attendance to the inclusion of surveyors fixed effect, N=373, Punjab, India

|  | **Follow-up Attendance** | | | |
| --- | --- | --- | --- | --- |
|  | **ITT** | | **ToT** | |
|  | (1) | (2) | (3) | (4) |
| **Reminder Effect** | 0.022  (0.041) | 0.021  (0.042) | 0.026  (0.051) | 0.026  (0.052) |
| **Facility Fixed Effect** | YES | YES | YES | YES |
| **Surveyors Fixed Effect** | NO | YES | NO | YES |
| **Control Mean** | 0.196 | 0.196 | 0.196 | 0.196 |
| **N** | 373 | 373 | 373 | 373 |

*Notes*: Coefficients are expressed as percentage points. Robust standard errors in parentheses. Surveyor information was recorded starting from July 29th, 2022, eleven days after the trial began. Therefore, we lack surveyor information for 15 (3.9%) participants. * p<0.1, ** p<0.05, ***p<0.01.

# Table A10 - Robustness of intention to treat and treatment on treated reminder effects on endline misconception score to the inclusion of surveyors fixed effect, N=346, Punjab, India

|  | **Misconception Score** | | | |
| --- | --- | --- | --- | --- |
|  | **ITT** | | **ToT** | |
|  | (1) | (2) | (4) | (5) |
| **Reminder Effect** | -0.194  (0.147) | -0.221  (0.152) | -0.233  (0.177) | -0.269  (0.184) |
| **Facility Fixed Effect** | YES | YES | YES | YES |
| **Misconception Score at Baseline** | YES | YES | YES | YES |
| **Surveyors Fixed Effect** | NO | YES | NO | YES |
| **Control Mean** | 2.101 | 2.101 | 2.101 | 2.101 |
| **N** | 346 | 346 | 346 | 346 |

*Notes*: Misconception score ranges from 0 to 7 and the corresponding coefficients are in units. Of the 388 participants composing the final sample, 386 were contacted to conduct the endline survey. Due to an implementation mistake, we failed to contact 2 (0.5%) participants. Of the remaining 386, 12 never picked up the phone (3.1%) and 14 (3.6%) ended the call before answering the seven false belief questions. Surveyor information was recorded starting from July 29th, 2022, eleven days after the trial began. Therefore, we lack surveyor information for 15 (3.9%) participants. Robust standard errors in parentheses. * p<0.1, ** p<0.05, ***p<0.01.

#

# Table A11 - Intention to treat and treatment on treated reminder effects on each question composing the endline misconception score, Punjab, N=360, India

|  | **ITT** | **LATE** |
| --- | --- | --- |
|  | **(1)** | **(2)** |
| **Question 1** |  |  |
| Reminder Effect | 0.115**  (0.045) | 0.138**  (0.053) |
| **Question 2** |  |  |
| Reminder Effect | -0.011  (0.051) | -0.013  (0.061) |
| **Question 3** |  |  |
| Reminder Effect | -0.011  (0.038) | -0.013  (0.045) |
| **Question 4** |  |  |
| Reminder Effect | 0.055  (0.044) | 0.066  (0.052) |
| **Question 5** |  |  |
| Reminder Effect | 0.021  (0.053) | 0.026  (0.063) |
| **Question 6** |  |  |
| Reminder Effect | 0.005  (0.026) | 0.006  (0.031) |
| **Question 7** |  |  |
| Reminder Effect | 0.019  (0.040) | 0.023  (0.048) |
| **Facility Fixed Effect** | YES | YES |
| **Misconception Score at Baseline** | YES | YES |
| **N** | 360 | 360 |

*Notes*: Misconception score ranges from 0 to 7 and the corresponding coefficients are in units. Of the 388 participants composing the final sample, 386 were contacted to conduct the endline survey. Due to an implementation mistake, we failed to contact 2 (0.5%) participants. Of the remaining 386, 12 never picked up the phone (3.1%) and 14 (3.6%) ended the call before answering the seven false belief questions. Robust standard errors in parentheses. * p<0.1, ** p<0.05, ***p<0.01.
